# Supplementary material for: New Late Pleistocene age for the Homo sapiens skeleton from Liujiang southern China
Source: Nat Commun. 2024 Apr 29;15:3611. doi: 10.1038/s41467-024-47787-3 (PMC11058812; doi:10.1038/s41467-024-47787-3)
Supplement: Supplementary file 1 — Supplementary Information [file 41467_2024_47787_MOESM1_ESM.pdf]

Supplementary Materials for

# New Late Pleistocene Age for the *Homo sapiens* skeleton from Liujiang southern China

Junyi Ge†, Song Xing†, Rainer Grün, Chenglong Deng, Yuanjin Jiang, Tingyun Jiang, Shixia Yang, Keliang Zhao, Xing Gao, Huili Yang, Zhengtang Guo, Michael D. Petraglia*, Qingfeng Shao*

†These authors contributed equally to this work
*Corresponding authors. qingfengshao@njnu.edu.cn (Q.F.S.)
m.petraglia@griffith.edu.au (M.D.P.)

This document includes:

Supplementary Materials and Methods
Figs. S1 to S14
Table S1 to S3
References 1-42

# Materials and Methods

## Contents

1. Discovery and previous studies
    1.1 Discovery of the Liujiang human fossils
    1.2 Previous dating attempts
2. Geographic setting and stratigraphy
3. Provenance constraints of the Liujiang human fossils
    3.1 Grain-size analysis
    3.2 Colour measurements
    3.3 Geochemical analysis
4. Radiocarbon dating
5. U-series dating analyses
    5.1 U-series dating of carbonates
    5.2 U-series dating of fossil bones and teeth
6. Optically stimulated luminescence (OSL) dating on the sediments
    6.1 OSL dating
    6.2. Bayesian age modelling

## 1. Discovery and previous studies

### 1.1 Discovery of the Liujiang human fossils

The Liujiang human fossils were discovered in a small cave, named "Tongtianyan", in the Liuzhou City, Guangxi Province in 1958, by a workman when digging sedimentary deposits for fertilizer. A complete fossilized modern *Homo sapiens* cranium was first discovered at more than ten meters from the cave entrance. Soon afterwards, some parts of the skeleton, including the thoracic vertebrae, lumbar vertebrae, sacrum, right hip, and femur, were unearthed one after another near the

cranium. It was suggested that these human fossils were all derived from the same adult male individual[17]. Meanwhile, some well-preserved mammal species fossils which were interpreted as part of the late Middle Pleistocene *Ailuropoda-Stegodon* fauna in South China[1,2] were also found. After the fossils were discovered, paleoanthropologists from the IVPP (the Institute of Vertebrate Paleontology and Paleoanthropology, Chinese Academy of Sciences) immediately travelled to the Tongtianyan cave for investigation. Unfortunately, since the fossil-bearing sediment had been mined for fertilizer, any detailed information about the exact site and stratigraphy where the human and mammalian fossils have been found was lost and thus remained ambiguous. The poor provenience[2,3] impeded accurate dating, which in turn severely undermined the evolutionary significance of the human fossils.

### 1.2 Previous dating attempts

Although the provenance was uncertain, some scholars tried to date Liujiang human fossils. Firstly, Yuan et al.[4] conducted U-series dating on some mammal fossils pieces collected from the Tongtianyan cave by staff members of the Liuzhou Museum in Guangxi Province. The results showed that the fossils were all older than 100 ka, which were basically consistent with the previous speculations on the faunal assemblages. Meanwhile, they also carried out a U-series analysis on the most significant second layer of well-developed flowstone in the sediment sequence and obtained an age of 67 ka[5]. Their results suggested that the Liujiang human fossils could be much older than this age estimate, when considering the other faunal assemblages found in the cave.

Later, Shen et al.[5] and Wang et al.[6] made further investigations on the stratigraphy. Based on field observations, they suggested that the cave deposit can be divided into four sedimentary units in an attempt to reconstruct the cave deposition process. They concluded that the human fossils came from Unit III, i.e., gravel sandy clay layer, based on the previous speculative descriptions by Wo et al.[3] and Pei et al.[2]. By dating the flowstone layers and flowstone fragments in the Liujiang sequence, Shen et al.[5]

and Wang et al.[6] concluded that the Liujiang human fossils were likely as old as ~111-139 ka, and possibly even older than ~153 ka.

## 2. Geographic setting and stratigraphy

The Tongtianyan cave (24°10′59″N, 109°25′56″E, 164 m above mean sea level) is situated on the south slope of a karstic mountain in southeastern Liuzhou city, Guangxi Province (Fig. 1). Metamorphic limestone of the Permian Qixia Formation covers a large part of the region, into which karst landforms developed because of the regional hydrological system. The Tongtianyan is a cave system developed in the Permian metamorphic limestone, with its entrance oriented roughly northeast-southwest and lies ~ 90 m above the Liujiang riverbed. Behind the entrance, a 15-m-long narrow passage leads to an open chamber, with its ground surface plane inclining toward the chamber, with a dip angle of ~10°, which facilitated the transportation of the sediments into the chamber and infilled the deep cave due to its abruptly increased depth there. On the front side of the chamber, there is a short straight tubular passage. At the left rear of the cave (Fig. 1c), there are two parallel long passages, named the North and South branches, which are connected at their ends. Past that there are numerous much narrower passages.

Most of the sedimentary deposits in Tongtianyan were preserved in the chamber and the North branch where a deep pit developed, with the maximum thickness of cave deposits over 4 m. Most of the fossil-bearing deposits in the cave were damaged by the local people and are now only partially observable in the front chamber. The human fossils are thought to originate from sediments near the entrance of the North branch, these sediments were almost cleared out. While the mammalian fossils can still be found *in situ* in the remnants of the sedimentary sequence in the chamber and occasionally in the disturbed deposits of the middle part of the North branch.

As result of fertilizer extraction by the local farmers in the last century, a north-south oriented stratigraphic section containing an almost complete sedimentary sequence is exposed in the back part of the chamber. An examination of this section and residual sediments attached to the cave walls in the front part of the North branch, shows that these strata are almost comparable. The Tongtianyan stratigraphic sequence can be divided into three major sedimentary units (Fig. 2, Supplementary Figure 1a). Unit I comprise a light grey tilt-bedded fine sand interbedded with thin layers of yellowish clay and has a thickness of over than 1 m (Supplementary Figure 1b), which can be only observed in the deep cave. This unit is mainly exposed beneath Unit II and covers the cave floor[5,6] and was named as "Lower Unit" by Shen et al.[5] and Wang et al.[6]. It represents the earliest allochthonous sediments supplied by surface or sub-surface stream systems outside the cave. Units II and III lie unconformably above. Unit II consists primarily of yellowish-brown bedded calcareous clay/silty clay and silty clay interbedded with thick gravel layers. Gravels from this unit are mainly limestone, mostly angular or sub-angular, and poorly sorted. This unit is mainly exposed along the north side of the cave, with a thickness of ~3.6 m. In Shen et al.[5] and Wang et al.[6], Unit II and some parts of the Unit III overlying this unit, are defined as the "Middle Unit". Within this unit, four flowstone layers, FL 1, FL 2, FL 3 and FL 4, are exposed, and the uppermost FL 1 bends and caps in an L-shape over the upper part of Unit II. FL1 truncated the lower flowstone layers FL2 and FL3 and welded with them, suggesting a long period of erosion prior to the deposition of Unit III. On the south side of the cave, a small amount of this unit can also be found along the cave wall. By dating the flowstone layers, Shen et al.[5] showed that they were contemporaneous, providing clear evidence for the large-scale erosive event. In this unit, some well-preserved mammal species fossils including *Ailuropoda melanoleucus, Rhinoceros sinensism, Stegodon orientalis, Megatapirus* sp., *Sus* sp., *Bovidae* and *Cervidae* may have ever been discovered. Unit III subsequently filled the gully developed in Unit II with part of its upper layers unconformably overlying Unit II (Supplementary Figure 1). Unit III consists mainly of brown gravel and

yellowish-brown clay, is about 3.8 m thick, and can be divided into five layers (from top to bottom) (Supplementary Figure 1a):

Layer 1: a flowstone layer with its thickness (2-15 cm) gradually increasing towards the northern end which consists of highly pure calcite and muddy carbonate (Supplementary Figure 1c).

Layer 2: reddish-brown clay layer with some thin beddings locally observable (60-100 cm). An obviously undulating unconformity is observable between this layer and the overlying Layer 1.

Layer 3: a yellowish-brown homogenous silty clay layer, on top of which is a thin layer of flowstone (15-40 cm) indicating a depositional hiatus prior the deposition of the Layer 2.

Layer 4: a consolidated pale yellowish-brown calcareous-cemented silty clay layer containing a few limestone breccia and flowstone fragments especially close to the boundary with Layer 3. Layer 4 terminates gradually northward, suggesting a small-scale erosion-filling event (20-35 cm).

Layer 5: a yellowish-brown sand gravel layer with a thickness of over 2 m, containing grey-brown angular and poor-sorted gravels cemented by a matrix of sandy clay. These gravels are dominated by limestones and flints, with grain sizes ranging from 1 to 10 cm, indicating an infilling event by the swiftly flowing water from outside the cave possibly associated with palaeoclimate events with high precipitation.

Thereafter, the variations of sediments from bottom to top in the sedimentary sequence of Unit III indicates a gradual change in the depositional environment from high-energy fluvial or gravitational deposition to low-energy hydrostatic deposition. The energetic water flow from outside the cave responding to the relatively wet/warm palaeoclimates with high precipitation during the early MIS 3 may led to the high supply of coarse sediments in the lower Layers 5 and 4, while the gradual cooling and

drying of the climate in the late MIS3 may also be an important reason for the weakening of hydrodynamics. Of course, the gradually decrease of depth drop from the passage behind the entrance to the chamber may be also responsible for the leveling down of the energy for sediment transportation as the sediments infilled in the cave.

In addition, comparing our stratigraphy divisions with that by Shen et al.[5] and Wang et al.[6], it can be observed that the sediment sequence comprised by Layers 3, 2 and 1, apart from the part overlying the Unit II, obviously corresponds to the "Upper Unit" in their studies. While the Layers 5 and 4, were divided as the "Instrusive Breccia" layer by them[5,6].

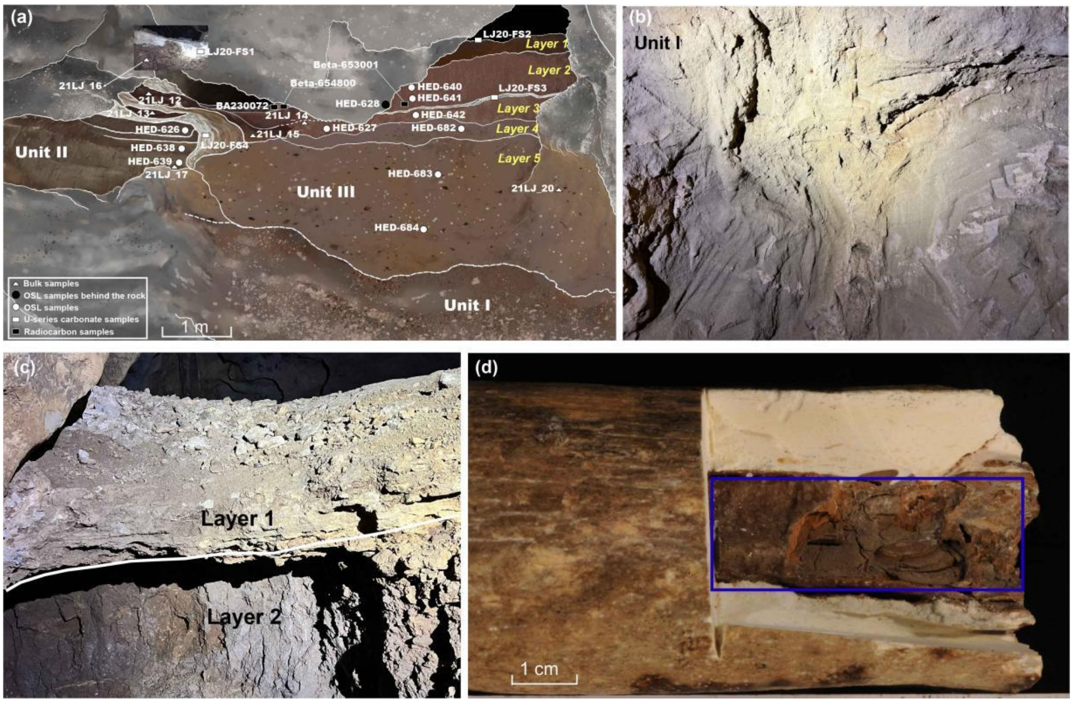


Supplementary Figure 1. The cave sketch shows locations of theses samples for radiocarbon dating (black rectangles), U-series dating (white rectangles), OSL dating (circles) and grain-size and geochemical analysis (circles and triangles) (a), and the sediments from Unit I (b) and Layer 1 in Unit III (c), as well as the sediments captured into the medullary cavity of the Liujiang femur (d). The blue rectangle marks the sampling position.

## 3. Provenance constraints of the Liujiang human fossils

### 3.1 **Grain-size analysis**

The particle contents and grain-size distributions of samples from different sedimentary layers are shown in Fig. 3c and Supplementary Figure 2. The size distributions of all samples are polymodal, mostly dominated by the silt fraction with very low proportions of particles > 63 μm (<1.5%) except for the two samples from Layer 4 and one from Unit II. Fitting the results with lognormal distribution functions suggests that the polymodal grain-size distribution of each sample comprises of between two and five unimodal distributions (Supplementary Figure 2a-d). Five types of multimodal distribution types can be identified. Samples from the Layer 2 of Unit III, such as the LJ_16, HED-640, HED-642 and HED-628, show rather similar bimodal distributions composed of two grain-size components of roughly similar proportions, and mode sizes of 0.6 μm and 3.65 μm, respectively. Sample HED-682 from Layer 4 also shows a similar bimodal distribution but having a smaller proportion of the silty mode size of 3.65 μm comparing with the samples from Layer 2. In Layer 3, four components with modal sizes of 0.18 μm, 1.42 μm, 7.18 μm and 30.32 μm, respectively, can be identified from the distributions of samples HED-627, HED-642, 21LJ-12, 21LJ-14 and 21LJ-15. These distributions are dominated by the modes with median sizes of 0.18 μm and 1.42 μm, with the finest modal having the highest proportion of 30-40%. In the Unit II, the sediments are almost unimodal with a strong peak around 7.18 μm of 6% and a smaller one around 0.6 μm of 2%, occasionally having a coarse component around 357.48 μm. The sediments from the Layer 5 of Unit III and the top part of the Unit II (21LJ-13) show a different type of multimodal distributions, characterized by a dominant broad modal peak around 3 μm, which is composed of four or five modes, and a minor coarse mode with the median grain-size of about 358 μm, suggesting the deposition by fluvial processes.

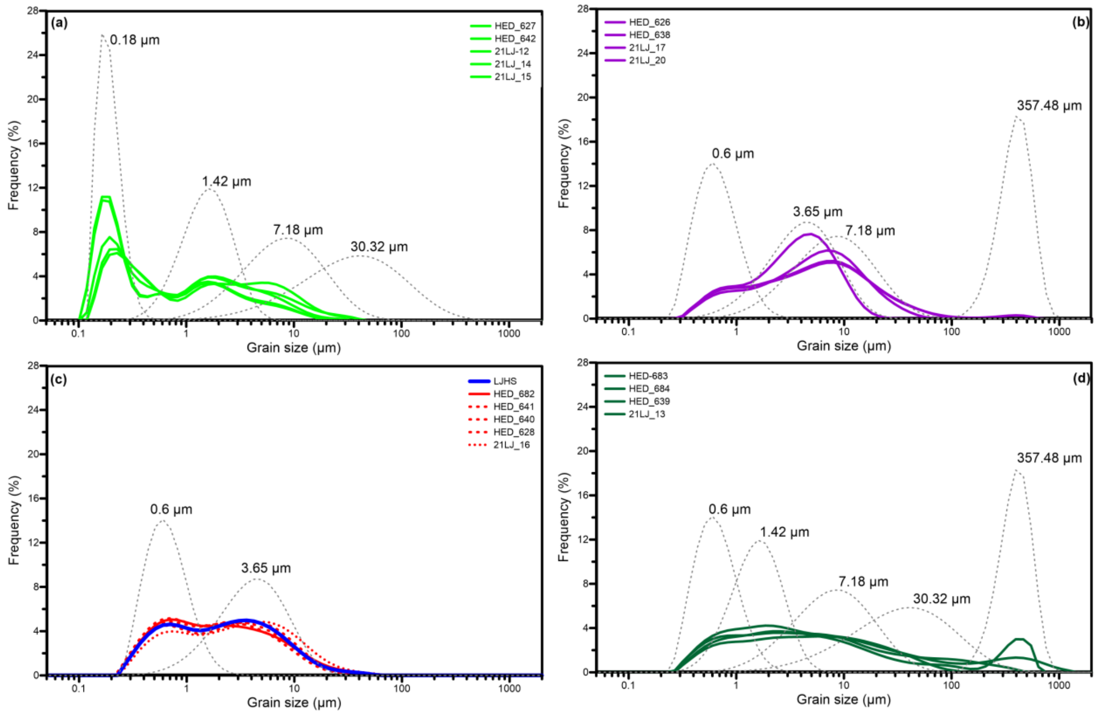


Supplementary Figure 2. Grain-size frequency distribution curves and grain-size components of samples from the sediments sealed in the human fossils and from the Tongtianyan stratigraphic sequence. The samples are grouped according to their similar grain-size distributions in (a)-(d). The curves in gray dashed lines represent the components fitted and partitioned within a polymodal distribution using the log-normal distribution function. The numbers labelled on these curves refers to the modal sizes. The grain-size plotted in x-axis is on a log scale. LJHS refers to the sediments trapped in the cavity of the Liujiang femur. Samples of HED-640, HED-641, HED-628 and 21LJ_16 are from Layer 2, HED-627, HED-642, 21LJ_12, 21LJ_14 and 21LJ_15 are from Layer 3, HED-682 is from Layer 4, HED-683 and HED-684 are from Layer 5 in Unit III, while samples of HED-626, HED-638, HED-639, LJ_13, LJ_17 and LJ_20 are collected from Unit II.

For the sediments caught in the in the cavity of Liujiang human femur, it shows a bimodal grain-size distribution quite similar with those of the samples from the Layer 2 of Unit III, suggesting that it might have derived from this layer.

In addition, for most of these layers or sedimentary units except for Layer 2, at least two samples were collected and analyzed. The samples from the same layer all show rather similar grain-size and size distribution, indicate good sediment homogeneity. Although the samples from Layer 5 have various percentage of coarse fraction, they also show similar grain-size distribution and mean grain-size varying from 6 μm to 4 μm, suggest that the fine sediments filled in the gravels also have relatively uniform particle composition.

### 3.2 **Colour measurements**

Variations of colour parameters or their ratios of sediments are mostly used as a tool for identifying a stratigraphic unit. Sediment colour measurement were conducted on the 19 samples used for grain-size analysis. The results are illustrated in the Fig. 3b and Supplementary Figure 3. It can be found that the sediments trapped in the hominin fossil (LJHS) show the similar a*, b* and L values with the samples from the Layer 2 of Unit III, especially with the HED-628 and HED-640 in the same level. However, LJHS has a different colour characteristics to HED-682 from the Layer 4, providing further evidence for our conclusion that the Layer 2 may be the human fossil-bearing layer.

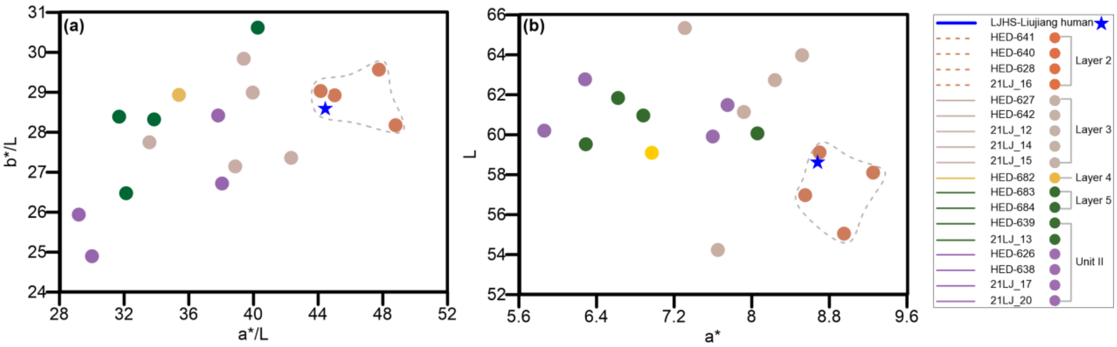


Supplementary Figure 3. Colour parameters from the cavity sediments housing the Liujiang human femur and from the Liujiang stratigraphic sequence. The sample of LJHS refers to the sediments trapped in the medullary cavity of the Liujiang femur. a* (L) refers to the redness (lightness) value of these sediments. a*/L and b*/L

correspond to the red-green and red-green scales calibrated by the lightness of sediments.

### 3.3 Geochemical analysis

The radiogenic isotopes of neodymium (Nd) and strontium (Sr) have proven to be reliable indicators of provenance because different source materials of different origins and ages generally have distinct Nd-Sr isotopic compositions, furthermore there is minor modification resulting from transportation, deposition and weathering processes[7].

Five representative samples were analyzed for trace element and Nd-Sr isotopic analysis, including the samples of LJHS and one each from Layer 2 (HED-628), Layer 3 (HED-627) and Layer 5 (HED-683), as well one from the Unit II (HED-626). Firstly, the silicate Nd-Sr isotopic ratios of the samples were determined by multi-collector inductively-coupled plasma mass spectrometer (MC-ICPMS) following the method of Chen at al.[8]. Secondly, the trace element compositions of these samples were determined using an ICP-MS (ELEMENT, Finnigan MAT). The samples were digested in $HNO_3$ and HF under high temperature and pressure using a two-step procedure as detailed in Ding et al.[9] in order to ensure complete refractory mineral dissolution. The acid-insoluble silicate fractions Sr-Nd isotopic compositions of the five samples are shown in Fig. 3a and Supplementary Table 1.

Supplementary Table 1. Sr and Nd isotopic composition of sediment samples from Liujiang Human fossil cavity (LJHS) and from the Liujiang section.

| Sample | $^{87}Sr/^{86}Sr$ | $^{143}Nd/^{144}Nd$ | $\varepsilon_{Nd}(0)$* |
|---|---|---|---|
| HED-626 | 0.719509 | 0.512214 | -8.26 |
| HED-627 | 0.723144 | 0.512212 | -8.31 |
| HED-628 | 0.728573 | 0.512213 | -8.29 |
| HED-683 | 0.711288 | 0.512275 | -7.07 |
| LJHS | 0.728238 | 0.512217 | -8.21 |

*$\varepsilon_{Nd}(0)=[(^{143}Nd/^{144}Nd)_{sample}/(^{143}Nd/^{144}Nd)_{CHUR}-1]\times 10000$; $(^{143}Nd/^{144}Nd)_{CHUR}=0.512638$.

Our results show that samples HED-626, HED-627, HED-628 and LJHS have similar $^{87}Sr/^{86}Sr$ ratios of 0.719509-0.728238, $^{143}Nd/^{144}Nd$ ratios of 0.512212-0.512217, and $\varepsilon_{Nd}$ values from -8.31 to -8.21. However, HED-683 from the Layer 5 shows much lower $^{87}Sr/^{86}Sr$ ratio (0.711288), and much higher $\varepsilon_{Nd}$ value (-7.07) and $^{143}Nd/^{144}Nd$ ratio (0.512275) (Fig. 3a and Supplementary Table 1), suggesting a different provenance of this layer.

Chemically immobile trace elements (e.g. rare earth elements (REE), Th, Zr, Co, Hf and Nb) have also proven to be of great potential for determining the provenance of clastic sediments[10-12], because they maintain invariant ratios during post-depositional chemical weathering. This approach has been used successfully to identify the origins of eolian deposits in various regions[13-16].

The results of trace element analyses (including REE) are listed in Supplementary Data 1. The chondrite-normalized REE distribution patterns are shown in Supplementary Figure 3, together with that of UCC (the Upper Continental Crust) for comparison. The sediment samples exhibit similar REE patterns, characterized by enriched light REE and relatively flat heavy REE profiles, and consistent with pronounced negative Eu anomalies (Supplementary Figure 3), which are similar to that of the UCC[12]. Some differences, however, occur between the sediment samples and UCC. Firstly, these samples including LJHS show pronounced negative Gd and positive La anomalies (see Supplementary Figure 4). Secondly, all sediments from the Liujiang section show pronounced negative Ce anomalies, but with the anomalies decreasing with distance from the carbonate layer (Layer 1) suggesting the effect by the carbonate. Generally, it has been observed that the carbonates in Southern China mostly show the negative Ce anomalies[17]. In addition, this anomaly was also found in the cave water drops in most caves in southern China[18]. Thirdly, the LJHS show no negative Ce anomalies at all, providing further support for our conclusion that this phenomenon was attributable to the post-depositional mixing of the carbonate carried

by the cave dripping or waterflow enriched carbonate. However, LJHS displays an enrichment of heavy REEs, possibly due to the influence from the fossils.

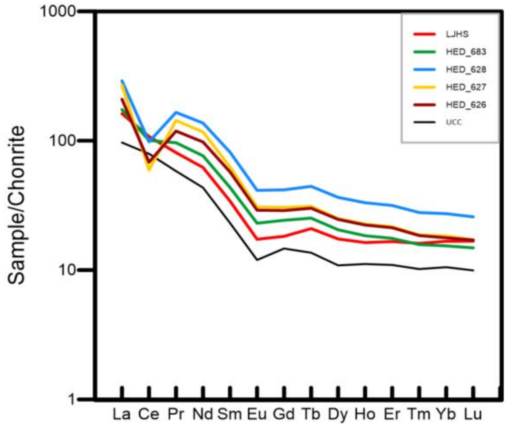


Supplementary Figure 4. Chondrite-normalized REE distribution patterns LJHS and sediment samples from the Liujiang section. UCC pattern is given as a reference. UCC and chondrite values are after Taylor et al.[12].

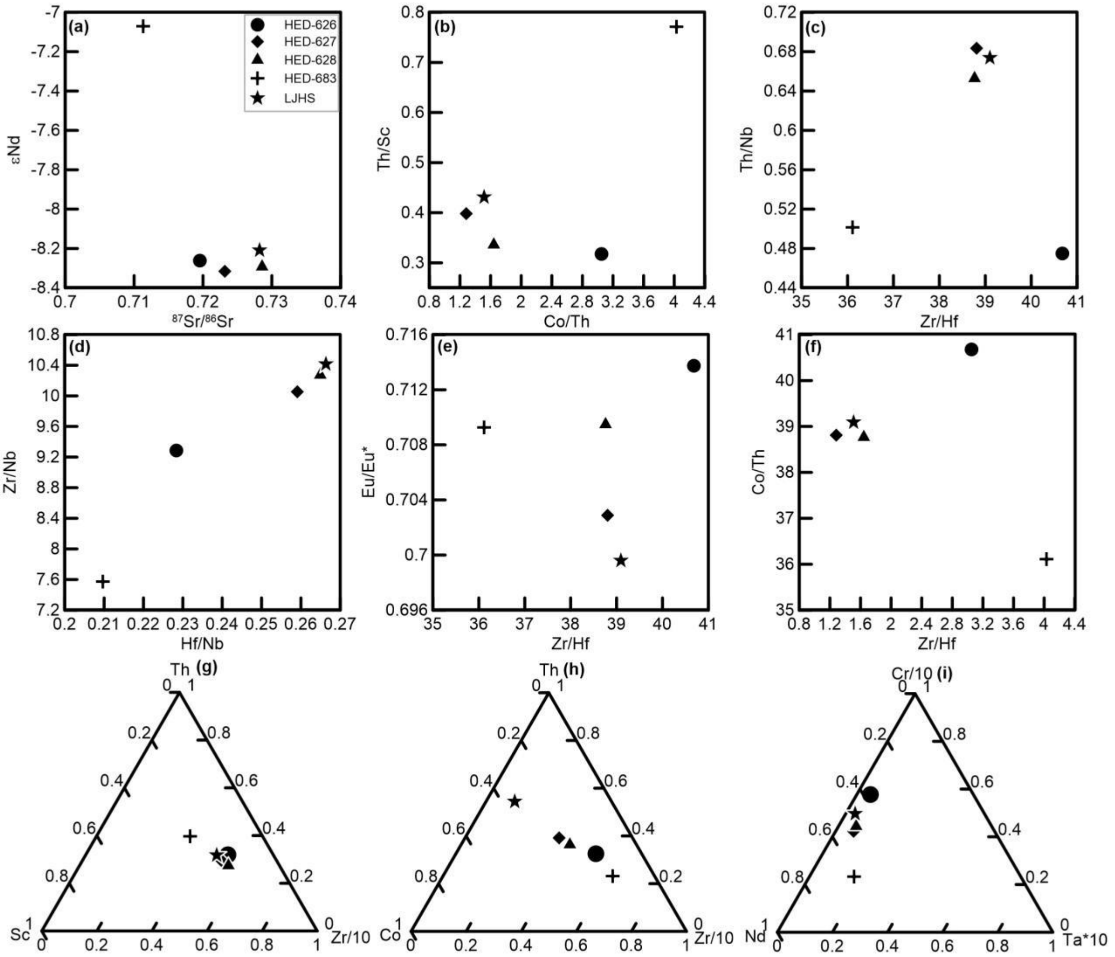


Supplementary Figure 5. Provenance diagrams. a-i, Provenance discrimination diagrams for the sediments from the cavity of the Liujiang human fossils and from the Liujiang stratigraphic sequence. The sample of LJHS refers to the sediments trapped in medullary cavity of the Liujiang femur.

The trace elements Ta, Y, Th, Sc, Zr, Co, Hf and Nb are thought to be most suitable for provenancing because of their relatively low mobility during sedimentary processes[102]. These elements are incorporated into clastic sedimentary rocks during weathering and transportation with little alteration and thus would reflect the signature of the parent material[10,19]. Generally, the ratios of Th/Sc, Co/Th, Zr/Hf, Th/Nb, Zr/Nb and Eu/Eu* are frequently used in sediment source discrimination[13,20]. Using these trace elemental ratio plots (Extended data Fig. 1b-f), LJHS can be clearly

separated from the sediments from the Unit II (HED-626) and the Layer 5 in the Unit III (HED-683), suggesting their different provenance. In contrary, LJHS shows similar results as HED-628 (Layer 2) and HED-627 (Layer 3). In ternary diagrams of Th-Sc-Zr/10, Th-Co-Z/10 and Nd-Ta*10-Cr/10 (Supplementary Figure 5g-i), which have been used successfully in the provenance identification of sediments[11-13,21], these differences can be also seen, confirming that the Layers 2 and 3 in the Unit III are the potential sediment source for the LJHS. Combining the evidence of the grain-size distribution and sediment colour analysis, we conclude that the Layer 2 in the Unit III represents the most likely location where the Liujiang human fossils have been unearthed.

## 4. Radiocarbon dating

The charcoal sample (BA230072) from Layer 1 yielded an age of 13.1-12.9 ka, while the sediment sample (Beta-654800) collected from the same layer but with slightly lower depth dated to 13.5-13.4 ka (Supplementary Table 2), agreeing with the age of the charcoal sample. The small discrepancy between the two dates possibly suggests minor effect of the contamination by old carbon preserved in the cave. The organic sediment sample from the Layer 3 gave an age of 30.4-30.0 ka, which high likely represent the depositional age of the sediment. This age is consistent with the OSL ages yielded by the samples in the same layer at 95.4% CI interval (see the OSL dating results below), which also provides supports for our conclusions.

Supplementary Table 2. AMS $^{14}C$ dating results.

| Laboratory code | Layers | Sample Type | Conventional $^{14}C$ age (yrs BP) | Error (yrs BP) | Calibrated age range (95.4% confidence, cal yrs BP) |
|---|---|---|---|---|---|
| Beta-654800 | 1 | *Organic sediment* | 11610 | 30 | 13516-13404 |
| BA230072 | 1 | *Charcoal* | 11135 | 50 | 13124-12915 |
| Beta-653001 | 2 | *Organic sediment* | 25920 | 120 | 30382-29969 |

## 5. U-series dating analyses

### 5.1 U-series dating of carbonates

The capping flowstone in Unit III, Layer 1 previously, dated to ~4–20 ka[5,6], provides a minimum age bracket for the underlying reddish clay layers. In this study, two samples from this layer were collected (Supplementary Figure 6a, b), yielding ages of 13.9 ± 0.7 ka and 16.1 ± 0.9 ka, in agreement with the previous dating results. All isotope ratios in this section are activity ratios.

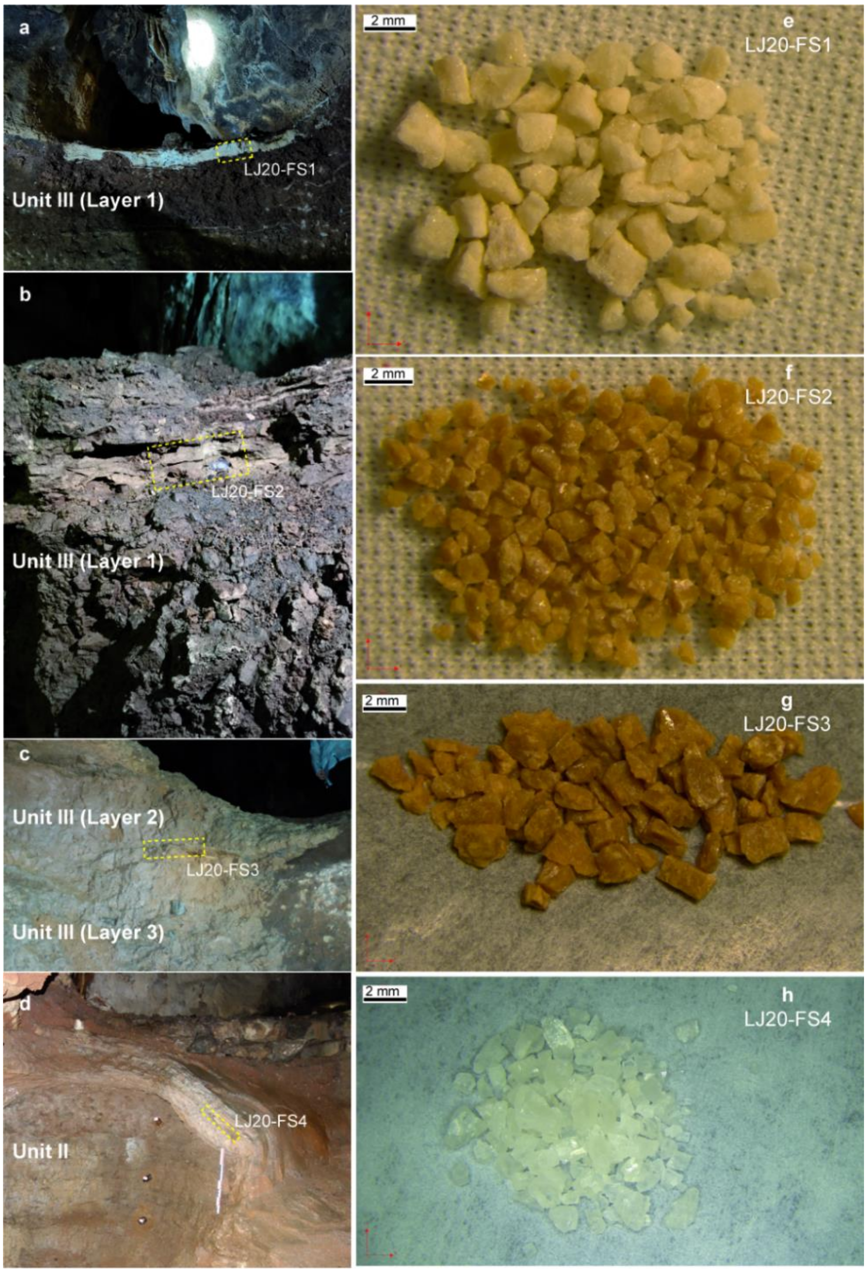

Supplementary Figure 6. Flowstone samples from Tongtianyan cave for U-series dating. a-d, Field photos for flowstones LJ20-FS1, -FS2, -FS3 and -FS4 (before sampling); e-h, The cleaned carbonate grains of samples LJ20-FS1, -FS2, -FS-3, and -FS4 for U-series dating.

In addition, a flowstone layer bracketed between the Layer 2 and 3 in Unit III, with varying thickness from 1 to 5 cm, was also sampled for U-series dating (Supplementary Figure 6c). Due to the low of $^{230}Th/^{232}Th$ ratios (Supplementary Data 2), we had to analyze 6 subsamples to determine the detrital-corrected $^{230}Th/^{238}U$ and $^{234}U/^{238}U$ ratios using the Rosholt isochron method. We obtained the detrital-corrected $^{230}Th/^{238}U$ ratio of 0.337 ± 0.056 and a $^{234}U/^{238}U$ ratio of 1.145 ± 0.10 (Fig. 5), which lead to an isochron age of 37.8 ± 8.6 ka for this flowstone (Supplementary Figure 7). This age represents a *terminus post quem* of Layer 2. The thick flowstone on the top of Unit II was previously dated to 61 ± 1 and 68 ± 1 ka with TIMS technique[3], representing the minimum age of the underlying deposits. We sampled a sample from the middle part of the flowstone (Supplementary Figure 6d) and obtained a U-series age of 63.07 ± 0.33 ka, confirming the previous results.

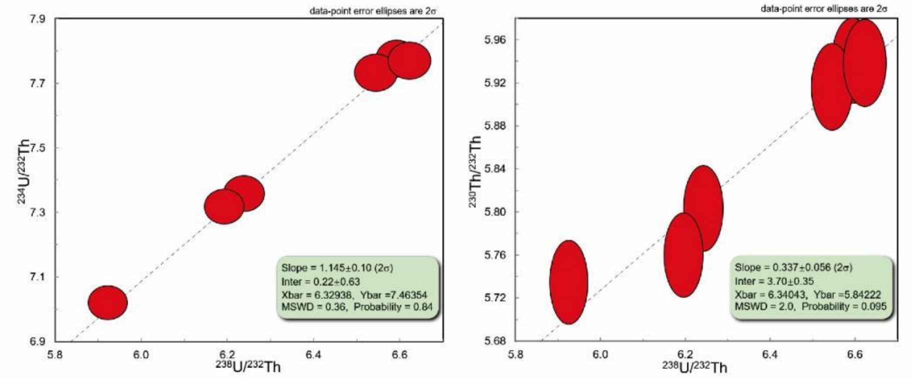


Supplementary Figure 7. Rosholt type-II isochron diagrams for the carbonate samples (LJ20-FS3) from the flowstone located at the interface of Layers 2 and 3 in Unit III. The isochron analyses were performed on the ISOPLOT program[26].

### 5.2 U-series dating of fossil bones and teeth

U-series dating was performed totally on 20 subsamples from Liujiang human fossils. In 2019, we tested U-series dating of the Liujiang human fossils using two dentine fragments collected from the container of the Liujiang cranium, which naturally separated from the upper central incisor as indicated by the attached resin glue on the outer surfaces (Fig. 5a-1), as well as bone fragments from the left femur (Fig. 5a-2, a-3), which were a byproduct of a previous sample extraction for ancient DNA and $^{14}C$ analyses.

Our first U-series analyses show that the dentine and bone samples are highly enriched in uranium (~56–96 μg/g), which is a typical phenomenon occurred in tooth/bone fossilization process. The dentine sample (LJ19-Dentine) yielded an apparent age of ~18.7 ± 0.1 ka, and the two sub-samples of the bones (LJ-Bone-A, -B) yielded slightly older apparent ages of 22.85 ± 0.04 ka and 21.64 ± 0.04 ka, respectively. Following the first attempt, two parallel profiles consisted of nine and eight subsamples (LJ20-1 to 9 and LJ20-(1) to (8)), respectively, were hand-drilled sequentially from the inner to the outer surfaces on the cortical bone section (~6 to 7 mm thick, Fig. 5a-5, a-6) using 0.3 mm carbide-tipped drill bits (~0.2–0.5 mg for each sub-samples) in 2020, to establish a reliable minimum age of the hominin fossils. In addition, eight newly discovered mammalian teeth were also analyzed, to test whether the Liujiang human fossils were associated with the *Ailuropoda-Stegodon* fauna remains. Among them, the sample LJ20-FT1 was found in the sediments adhering to the cave ceiling (Supplementary Figure 8 and Supplementary Data 2), and the others (LJ20-FT2 to 8) were found in the disturbed cave sediments, probably left when mining cave deposits for fertilizer (Supplementary Figure 8b, d-j).

U-series analytical results on the two parallel profiles are presented in Fig. 5, and Supplementary Data 2. The U-concentrations vary between ~30 and 60 μg/g in the

first profile (LJ20-1 to -9), and between ~30 and 80 μg/g in the second profile (LJ20-(1) to (8)). The high precision U-series analyses reveal that both of $^{234}U/^{238}U$ and $^{230}Th/^{238}U$ activity ratios display U-shaped distribution, though the $^{230}Th/^{238}U$ patterns are less symmetric than that of the $^{234}U/^{238}U$ (Fig. 5). The U-series apparent ages vary in the ranges of 21.39 ± 0.06 to 23.00 ± 0.08 ka and 21.55 ± 0.06 to 23.25 ka, for the two profiles, respectively, and following the distribution of $^{230}Th/^{238}U$ (Fig. 5). These isotopic patterns can be explained as U-uptake from the inner and outer surfaces towards the interior portions by the mechanism of diffusion and absorption, i.e, the D-A model[27,28]. The D-A model was recently refined by the DAD (diffusion-adsorption-decay) model[29], which takes in account the decay of $^{234}U$ through U diffusion. The U-uptake process of the Liujiang femur was thus modeled with the DAD model using the iDAD program[29]. The two profiles yielded consistent results: maximum likelihood burial ages of 23.8 ± 0.7 and 24.0 ± 0.8 ka with initial $^{234}U/^{238}U$ ratio of 1.192 and 1.191 and D/R of 9.27E-12 and 1.00E-11 $cm^2/s$. D/R is the diffusion-adsorption parameter for uranyl in bone, typically ranging from 1.0E-14 to 1.0E-12 $cm^2/s$[27]. D is the diffusion coefficient for uranyl in bone ($cm^2/s$). R is the volumetric equilibrium coefficient, the amount adsorbed per unit amount of solution. Large D/R ratios indicate fast diffusion and lead to shallow gradient of apparent U-series ages across a bone section. Small D/R values indicate slow diffusion and lead to steep gradients of apparent U-series age profile.

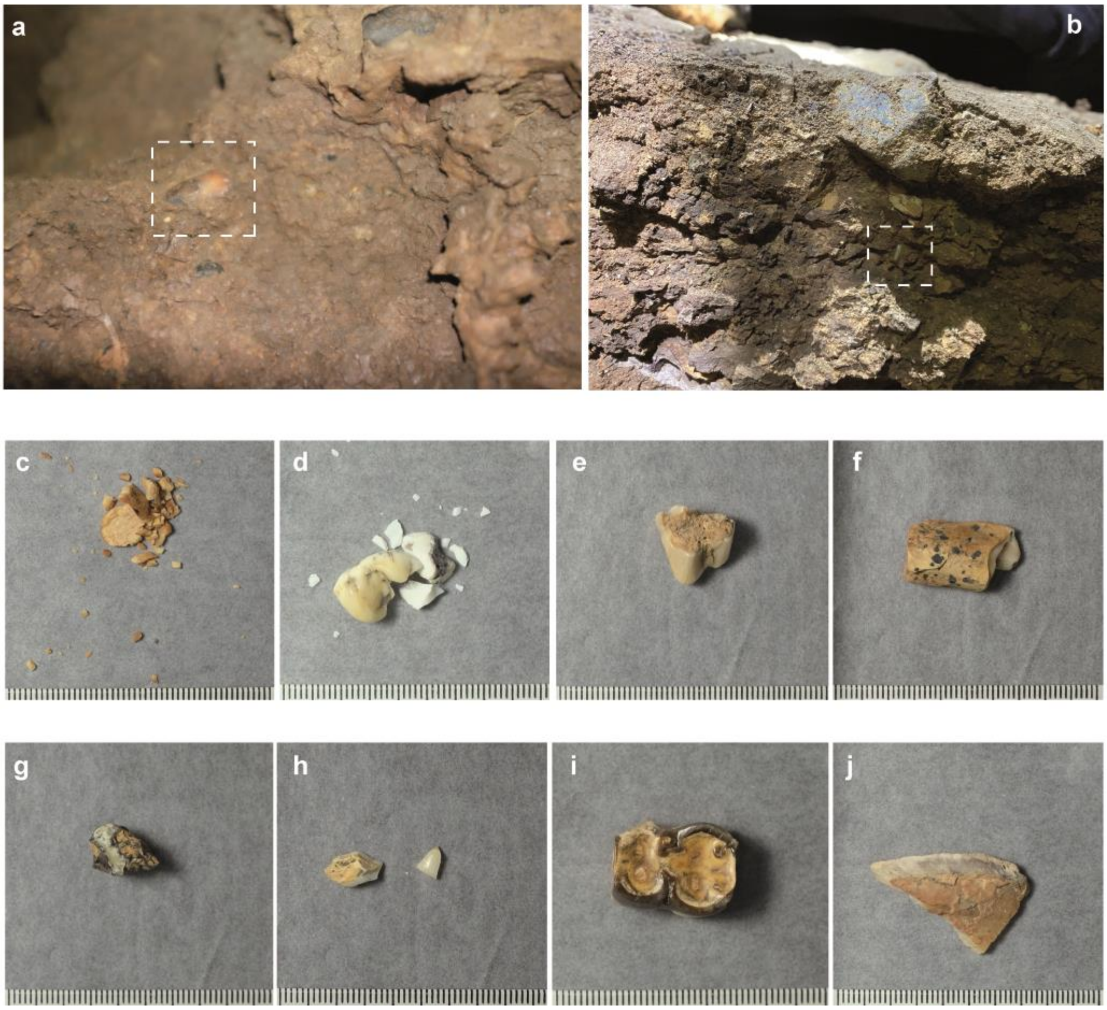


Supplementary Figure 8. Mammalian fossil teeth from Tongtianyan cave for U-series dating. a, Field photo for the fossiliferous sediments adhering to the cave ceiling (before sampling). b, Field photo for the disturbed fossiliferous sediments (before sampling). c-j, Tooth samples LJ20-FT1 to 8 found in 2020 (the minor graduation marks on the scale indicate 1 mm). The dashed white boxes in panel a and b highlight the presence of mammalian fossils in the cave sediments.

The U-series isotopic data of the newly found mammalian teeth fall into an age range from ~210 ka to 110 ka which are comparable with the previously published U-series ages of the Liujiang faunal data[5], but significantly older than the U-series dating results of the Liujiang human fossils (Fig. 5c-1, c-2, Supplementary Data 2). Fig. 5c shows that the human and mammalian samples are not only different in age but also in their initial $^{234}U/^{238}U$ ratios, ~1.2 vs >1.45. The initial $^{234}U/^{238}U$ ratios characterize the

source of the uranium that is postdepositionally incorporated into the skeletal material. This result again demonstrates that the Liujiang human fossils were buried in a distinctively different sedimentary environment to that of the mammalian remains.

## 6. Optically stimulated luminescence (OSL) dating on the sediments

### 6.1 OSL dating

Supplementary Table 3. The Single-aliquot Regenerative-dose (SAR) procedure used for $D_e$ determination in this study. In Steps 2 and 6, the aliquot is cooled to <60 °C after heating. $L_X$ ($L_N$) and $T_X$($T_N$) refer to the OSL response to a regenerative (Natural) dose and corresponding test dose OSL response, respectively, which are derived from the initial OSL signal (0.8 s) subtracting a background estimated from the last 4 s of the stimulation curve.

| Step | Treatment | Observed |
|---|---|---|
| 1 | Given dose, Di[a,b] (natural or laboratory) | |
| 2 | Preheat for 10 s at 260 °C | |
| 3 | IR stimulation for 40 s at room temperature [c] | |
| 4 | Stimulate for 40 s at 125 °C | $L_X$ |
| 5 | Test dose, Dt | |
| 6 | Heat to 220 °C | |
| 7 | Stimulate for 40 s at 125 °C | $T_X$ |
| 8 | Return to Step 1 | |

a :Repeated dose was given at last to monitor the efficiency of sensitivity change correction.

b :$D_0$=natural dose.

c :IR stimulation is conducted before blue stimulation in the last recycling step at room temperature.

Supplementary Figure 9 shows natural and regenerative decay curves for the representative samples HED-682 from Layer 4 in the Unit III and HED-626 from Unit II. They both exhibit typical decay curve of quartz minerals and OSL signal decreasing very quickly during the first second of stimulation. The OSL signals from the first 0.8 s of optical stimulation time is dominated by the fast component. The fast component from quartz is considered preferable for equivalent dose calculation in OSL dating[30-32]. The dose response curve of these samples (inset in Supplementary Figure 9) can be well-fitted by single saturating exponential fitting or by the sum of an exponential and a linear component using at least five regeneration dose points, including a zero-dose for the measurement of recuperation and a recycling point (blank diamond) for assessing the sensitivity change correction.

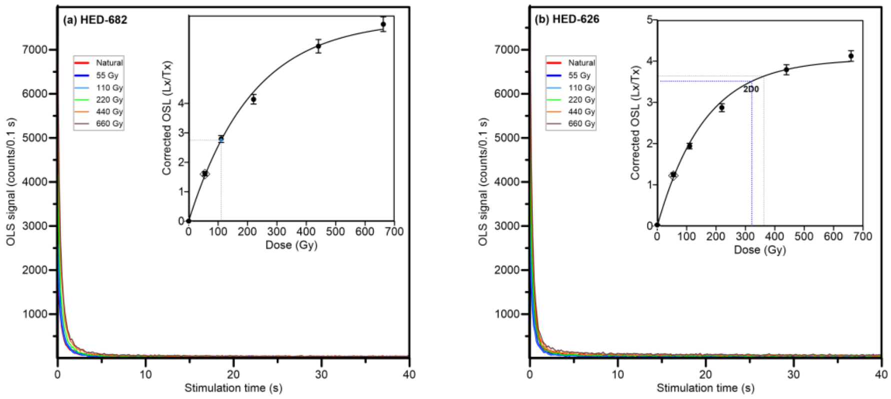


Supplementary Figure 9. Decay curves and dose response curves (inset) for samples HED-682 and HED-626. $L_x/T_x$ represents the sensitivity-corrected luminescence intensity to the test dose. Blue dashed line refers to the position of $2D_0$ value for the sample of HED-626.

The preheat plateau tests results are presented in Supplementary Figure 10. This figure shows that the obtained quartz $D_e$ values appear to be independent of varied preheat temperatures between 200 to 280°C, and the recuperation values are less than 5%. The weighted means of recycling ratios for all investigated preheat temperatures are mostly between 0.9 and 1.1, but systematically smaller than 1 (Supplementary

Figure 10), which possibly suggests a slight decrease in the shape of the dose response curve during successive measurement cycles, and seems to result in a slight overestimation of the equivalent doses (and, hence, the OSL ages). However, the recycling ratio at a preheat temperature of 260 °C is extremely close to unity (i.e., with a weighted mean of 0.99 and a standard error of 0.02). In addition, as shown in the results from dose recovery tests, no obvious overestimation of the given dose during the dose recovery test for these eight unsaturated samples when a preheat temperature of 260 °C was applied (as shown in the Supplementary Figure 11). It shows that the dose recovery ratios and the corresponding recycling ratios are overall within 10% of unity, with weighted means of 0.99 ± 0.05 and 0.99 ± 0.04, respectively (Supplementary Figure 11). The recuperation values are also generally lower than 5%. Therefore, a preheat temperature at 260° C with a cut heat at 220 °C was applied for the $D_e$ determination of all samples.

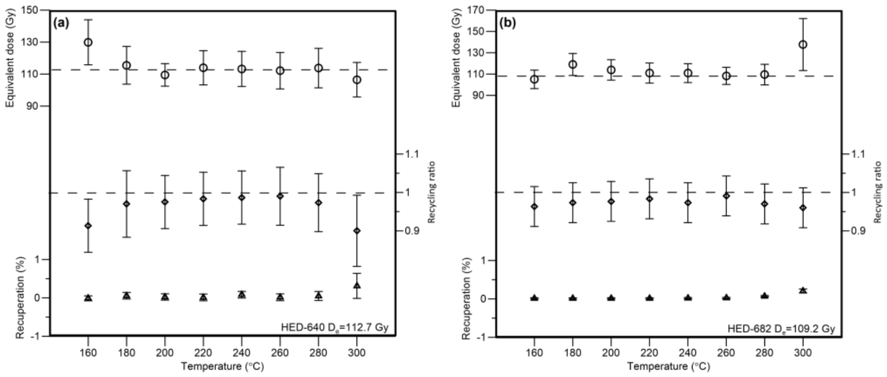


Supplementary Figure 10. Results of the preheat plateau test for samples HED-640 (a) and HED-682 (b). Each data point represents the average of three measurements.

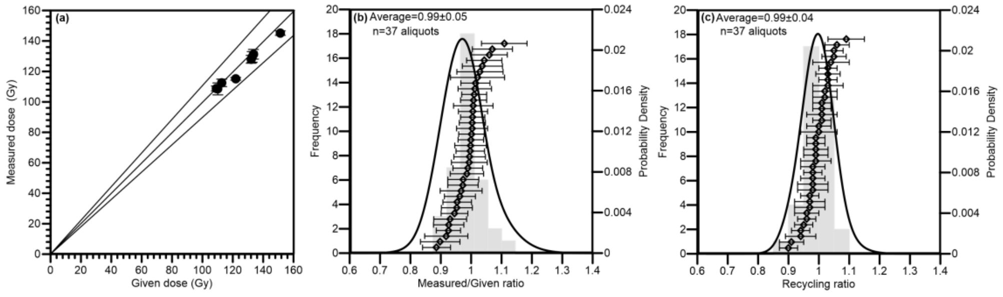

Supplementary Figure 11. Results of dose recovery shown as measured to given dose plots (a) and probability density plot (b), as well as histogram (in gray color) (b) for eight unsaturated samples from the site. The recycling ratios for dose recovery test of these eight samples using the preheat and cut-heat temperatures of 260 and 220 °C are also shown as probability density plot and histogram (in gray color) (c). All error bars represent 1 s.e.m.

The $D_e$ values of the aliquots with recycling ratios of <0.9 or >1.1, and with recuperation values >5% were excluded for $D_e$ determination. Finally, 14-40 aliquots were measured for $D_e$ determination of each sample.

$2D_0$ (86% of the saturation intensity for a single saturating exponential growth curve fitting) values is generally taken as the practical upper limit for dose estimation[32] to evaluated whether the OSL signals for each measured aliquot is saturated or not, and the aliquots with $D_e$ values higher than which was mostly used to calculate minimum ages. For all samples from the Unit III ($D_e$<160 Gy), no aliquot met the criterion. However, in the three samples including HED-626, HED-638 and HED-639 from the Unit-II, those measured aliquots all most yielded $D_e$ values higher than $2D_0$. Some studies suggested that reliable $D_e$ can be obtained when the shape of the growth curve can be accurately established and the untruncated natural signals are properly used for $D_e$ estimate[33], and it has also been proved that accurate $D_e$ estimates at higher doses[34-37] can actually be obtained by a by a function which is the sum of a single saturating exponential and linear component or by the sum of two saturating exponentials when the growth curves of the samples have an additional linear or exponential components. Thereafter, the data for these three samples, thereafter, were fitted with the sum of an exponential and a linear component, which seem to aligns with the regeneration data points better. Finally, the arithmetic mean was used for $D_e$ and age calculations for each sample, and an absolute standard error was also calculated for the uncertainty estimation (Supplementary Figure 9, Supplementary Data 4).

Incomplete resetting is an issue for OSL dating. However, signal bleaching seems not to be a problem for the samples from Tongtianyan cave, for the following reasons: (1) The sediments in Layers 2 and 3 are mainly dominated by homogeneous silty clay, suggesting that they may have been extensively weathered and transported into the cave by low-energy slope sheet flow rather than by flood-like water current. Given that the quartz mineral can be bleached in a few minutes, and the luminescence signals of quartz grains are dominated by the fast component, it therefore seems that these quartz grains may have experienced full exposure to sunlight. Afterwards, owning to the sloping ground surface toward the deep cave in the passage connecting the cave entrance with the chamber where the sediments accumulated, with a dip angle of ~10°, the sediments entering the cave can be transported smoothly to the deep chamber and trapped there without disturbance. As the deep cave where the sediments deposited is absolutely in darkness, there is no chance for resetting of the OSL signal for them, the burial age of the human fossils seems to be close to that of the resetting time of the luminescence signals outside the cave. (2) All (unsaturated) samples from the Unit-II in Tongtianyan cave typically describe a normal Gaussian distribution of $D_e$ values, with only a few samples slightly skewed (Supplementary Figure 12), and all give over-dispersion ($\sigma$OD) values representing the relative standard deviation of the $D_e$ distribution of <20%, indicating that these sample may have been well bleached prior to deposition[38,39]. (3) $D_e$ as a function of illumination time ($D_e$(t)), is also used to identify the partial resetting of quartz OSL signals[40]. As shown in the $D_e$(t) plots (Supplementary Figure13), all of the eight unsaturated samples yielded consistent $D_e$(t) values at the 1$\sigma$ confidence level independent of signal integration, which also well suggests full resetting of the fast and medium components prior to deposition[40]. In addition, the consistency between OSL ages and radiocarbon ages in the same layer also provides additional support for the conclusion that there is good OSL signal bleaching for the quartz from Tongtianyan cave.

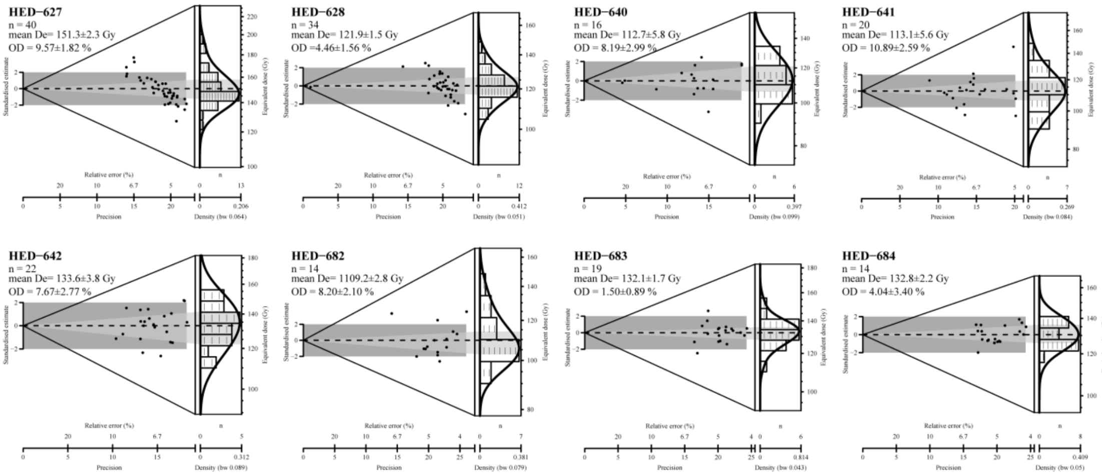


Supplementary Fig.12. Equivalent dose distributions of these unsaturated OSL samples from the Liujiang sequence.

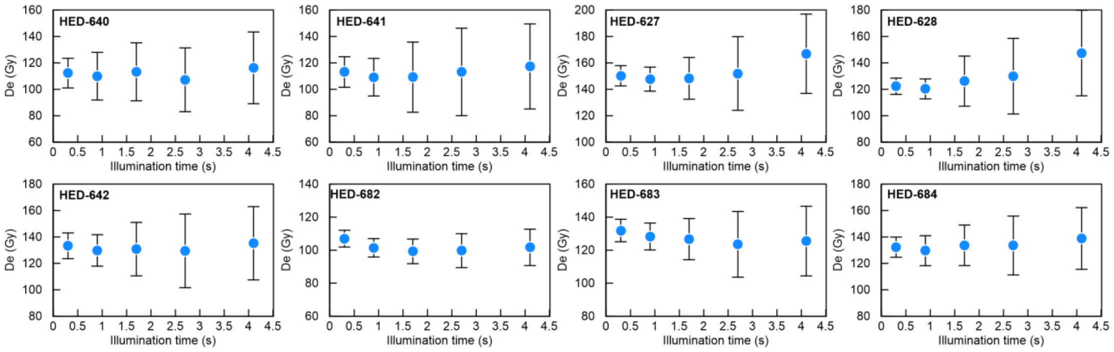


Supplementary Figure 13. $D_e(t)$ results for eight unsaturated samples from the Liujiang sequence, which are calculated using the intervals of 0-0.3s, 0.3-0.9s, 0.9-1.7s, 1.7-2.7s and 2.7-4.1 s, respectively, as suggested by Bailey et al.[40]

The concentration of natural $^{238}U$, $^{232}Th$, $^{226}Ra$ and their decay products, and $^{40}K$ for the OSL samples collected from the Liujiang sequence are shown in the Supplementary Data 3. The specific radionuclide activity ratios were plotted as Supplementary Figure 9 to examine the disequilibria on dose rate estimates for these samples. For most of samples from Unit III, the concentrations of $^{238}U$ are close to that of $^{226}Ra$ with a $^{238}U/^{226}Ra$ ratio ranging from 0.8 to1.0, indicating that they are within 20% of equilibrium. While for the three samples of HED-626, HED-638 and

HED-639 from Unit II, the $^{238}U$ and $^{226}Ra$ concentrations differ by more than 30%, even than 50%, indicating that $^{226}Ra$ concentrations apparently deficient with respect to $^{238}U$ in these samples. The $^{210}Pb/^{226}Ra$ activity ratios of all samples range from 1.0 to 0.8 at the 1σ confidence level, which is likely the result of the escape of the intermediate gaseous nuclide $^{222}Rn$[41]. All samples from Unit III show $^{210}Pb/^{238}U$ activity ratios ranging from 1.22 ± 0.06 to 0.77 ± 0.05, within 20% of equilibrium (activity concentration ratios of 0.8-1.2). While for these samples from Unit II, the $^{238}U$ concentration are at least 30% higher than that of $^{210}Pb$ radionuclide, which is similar with the difference between the $^{238}U$ and $^{226}Ra$ concentrations. The above results clearly show that a disequilibrium exceeding 30% is present in the $^{238}U$ decay chain for the sediments from Unit II in the Liujiang sequence, but with a lower level of less than 20% for the other sample from Unit III. Considering the thick layer of flowstone at the top of Unit II, we speculate that the downward leaching of parental $^{238}U$ during the flowstone formation may have responded to the high $^{238}U$ concentration in the three OSL samples from Unit II and subsequent disequilibria in the $^{238}U$ decay chain. These additional $^{238}U$ resulted from the downward leaching carbonate may have led to an overestimation of the total dose rate for the three sample HED-626, HED-638 and HED-6329 from the Unit II by 0.27 ± 0.27 Gy/ka, 0.94 ± 0.36 Gy/ka and 0.55 ± 0.26 Gy/ka in maximum, respectively, and thus caused an age underestimation of 4 ± 4%, 18 ± 8% and 15 ± 8%, respectively. Nevertheless, due to saturation of the luminescence signals for these samples from Unit II, the possible underestimation of the total dose rated resulted from the U-series disequilibria seem to have little effect on the OSL dating for these samples.

For all these eleven samples from Units II and III, both the two parent-daughter concentration pairs of $^{228}Th/^{228}U$ and $^{228}Ra/^{232}Th$, are with analytical uncertainty of equilibrium, with the activity ratio ranging from 0.96 ± 0.03 to 1.03 ± 0.03 and from 0.98 ± 0.02 to 1.01 ± 0.02, respectively, indicating that the $^{232}Th$ chain is at secular equilibrium for the sediments in the Liujiang sequence. Finally, given that the

disequilibrium in the U-series is observable in some samples, we calculated the dose rates and ages using the ager program by Murray et al.[42], in which the contribution of all parent and daughter radionuclides are fully included. In addition, to avoid the possible effect by inhomogeneity of the sediments and reduce the inaccuracies to gamma dose rate estimates for sediments deposited in caves, the gamma dose rates achieved by *in-situ* (field) gamma spectrometry measurements were combined with the water-corrected alpha and beta dose rates yielded by HRGS to calculate the OSL ages.

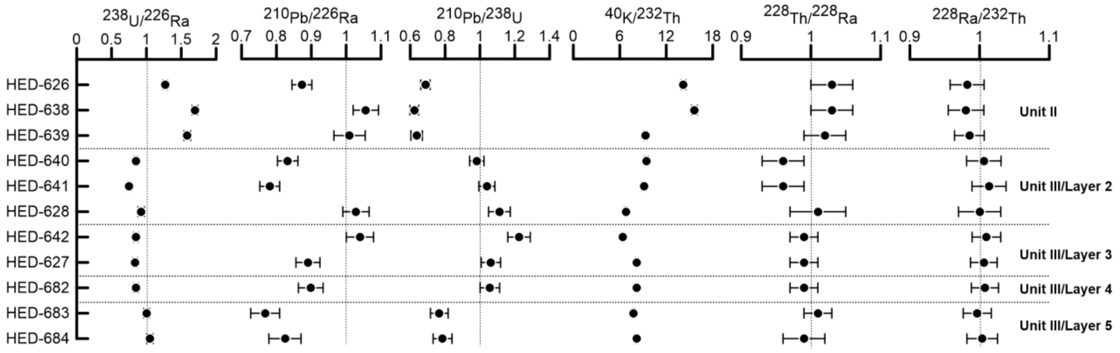


Supplementary Figure 14. Variations of specific radionuclide activity ratios for the OSL samples from the Liujiang sequence. The dash lines represent the condition of secular equilibrium (e.g. $^{228}Th/^{228}Ra=1$).

The results of dose rate calculation as well as OSL ages are listed in Supplementary Data 4, and OSL ages are also shown in Fig. 4b. Our results show that the samples from the Layer 2 yield the similar OSL ages, which fall into the narrow age ranges from ~25-22 ka and agrees well with the radiocarbon dates in 95% CI. The two samples constrain the age of the Layer 3 between ~38 ka and ~34 ka, and those from the Layers 4 and 5 generated an OSL age range spanning from ~53-42 ka. However, the three samples from the Unit-II beneath the thick flowstone layer (FL-2) generate the ages of 62 ± 4 ka, 103 ± 7 ka and 151 ± 12 ka, respectively, which are much older than the samples from Unit III. The samples HED-626 and HED-638 are collected between the flowstone Layer FL-2 and Layer FL-3, which were dated to 71 ± 5 ka and 133 ± 11 ka by carbonate U-series dating method, respectively. While the sample

HED-639 was taken from the layer under FL-3 but above the flowstone Layer FL-4 which has an age of 240 ± 37 ka. It can be seen that these ages agree well with our U-series dates as well as previously published U-Th dating results on these flowstone layers[5], even considering the possible age underestimation due to the U-series disequilibrium U-series disequilibrium in this unit.

### 6.2. Bayesian age modelling

The model suggests that deposition of the lowermost Layer 5 of Unit III, started at 52.2 ± 11.0 ka (at 95% CI) (Supplementary Data 5). The topmost Layer 1 has an age range from 16.1 ± 2.7 to 11.6 ± 1.6 ka, which is also consistent with the previously reported U-Th ages of 18 ± 2 ka to 13 ± 3 ka on northern part of this layer[5]. The Layers 3 and 2 span between 39.4 ± 6.3 and 37.2 ± 5.2 ka and from 32.5 ± 2.5 to 22.6 ± 7.4 ka at 95% CI, respectively. In addition, the age of the boundary between Layers 3 and 2 was modelled from 37.2 ± 5.2 ka to 32.5 ± 2.5 ka, which is also in consistence with the isochron age of 37.8 ± 8.6 ka yielded by the flowstone layer bracketed between the Layer 2 and 3.

## References


1 Huang, W. B. On the age of the cave faunas of South China. *Paleovertebrata et Paleoanthropologia* **17**, 327-341 (1979).

2 Pei, W. Z. in *Institute of Vertebrate Paleontology and Paleoanthropology, Academia Sinica* Vol. Memoir No. 7 1-54 (Science Press, 1965).

3 Wo, J. Human fossils found in Liujiang, Guangxi, China. *Paleovertebrata et Paleoanthropologia* **1**, 97-104 (1959).

4 Yuan, S. X., Chen, T. M. & Gao, S. J. Uranium series chronological sequence of some Palaeolithic sites in South China. *Acta Anthropologica Sinica* **5**, 179 (1986).

5 Shen, G. J. *et al.* U-Series dating of Liujiang hominid site in Guangxi, Southern China. *Journal of Human Evolution* **43**, 817-829 (2002).

6 Wang, W., Shen, G. J., Zhou, C. L., Wang, Q. & Zhao, J. X. Stratigraphy and chronology of deposits in Liujiang Hominid Cave, Guangxi, China. *Quaternary Sciences* **24**, 272-277 (2004).

7 Grousset, F. E. & Biscaye, P. E. Tracing dust sources and transport patterns using Sr, Nd and Pb isotopes. *Chemical Geology* **222**, 149-167 (2005).

8 Chen, J. *et al.* Nd and Sr isotopic characteristics of Chinese deserts: implications for the provenances of Asian dust. *Geochimica et Cosmochimica Acta* **71**, 3904-3914 (2007).

9 Ding, Z. L., Sun, J. M., Yang, S. L. & Liu, T. S. Geochemistry of the Pliocene red clay formation in the Chinese Loess Plateau and implications for its origin, source provenance and paleoclimate change. *Geochimica et Cosmochimica Acta* **65**, 901-913 (2001).

10 Bhatia, M. R. & Crook, K. A. Trace element characteristics of graywackes and tectonic setting discrimination of sedimentary basins. *Contributions to mineralogy and petrology* **92**, 181-193 (1986).

11 McLennan, S. M., Hemming, S., McDaniel, D. K. & Hanson, G. N. Vol. 284 *Processes Controlling the composition of clastic Sediments* (eds M.J. Johnsson & A. Basu) 21-40 (Geological Society of America, 1993).

12 Taylor, S. R. & McLennan, S. M. The continental crust: its composition and evolution. (1985).

13 Hao, Q. Z., Guo, Z. T., Qiao, Y. S., Xu, B. & Oldfield, F. Geochemical evidence for the provenance of middle Pleistocene loess deposits in southern China. *Quaternary Science Reviews* **29**, 3317-3326 (2010).

14 Muhs, D. R. *et al.* Geochemical evidence for airborne dust additions to soils in Channel Islands National Park, California. *Geological Society of America Bulletin* **120**, 106-126 (2008).

15 Reheis, M. C., Budahn, J. R. & Lamothe, P. J. Geochemical evidence for diversity of dust sources in the southwestern United States. *Geochimica et Cosmochimica Acta* **66**, 1569-1587 (2002).

16 Sun, J. M. Provenance of loess material and formation of loess deposits on the Chinese Loess Plateau. *Earth and planetary science letters* **203**, 845-859 (2002).

17 Liu, Q. Q. *et al.* Distribution and influencing factors of rare earth elements in carbonate rocks along three continental-scale transects in eastern China. *Earth Science Frontiers* **25**, 99-115 (2018).

18 Li, Y. L., Wang, S. J., Sun, C. X. & Liu, X. m. The characteristics of Ce anomalies in weathering crusts of carbonate rocks and its formation. *Journal of Mineralogy and Petrology* **25**, 85-90 (2005).

19 McLennan, S. M., Taylor, S. & Kröner, A. Geochemical evolution of Archean shales from South Africa. I. The Swaziland and Pongola Supergroups. *Precambrian Research* **22**, 93-124 (1983).
20 Xie, Y. Y. *et al.* Geochemistry of loess deposits in northeastern China: constraint on provenance and implication for disappearance of the large Songliao palaeolake. *Journal of the Geological Society* **175**, 146-162 (2018).
21 Garcia, D., Fonteilles, M. & Moutte, J. Sedimentary fractionations between Al, Ti, and Zr and the genesis of strongly peraluminous granites. *The Journal of Geology* **102**, 411-422 (1994).
22 Cheng, H. *et al.* Improvements in $^{230}$Th dating, $^{230}$Th and $^{234}$U half-life values, and U–Th isotopic measurements by multi-collector inductively coupled plasma mass spectrometry. *Earth and Planetary Science Letters* **371**, 82-91 (2013).
23 Holden, N. E. Total half-lives for selected nuclides. *Pure and Applied Chemistry* **62**, 941-958 (1990).
24 Goldstein, S., Murrell, M. & Janecky, D. Th and U isotopic systematics of basalts from the Juan de Fuca and Gorda Ridges by mass spectrometry. *Earth and Planetary Science Letters* **96**, 134-146 (1989).
25 Ludwig, K. & Titterington, D. Calculation of $^{230}$Th/U isochrons, ages, and errors. *Geochimica et Cosmochimica Acta* **58**, 5031-5042 (1994).
26 Ludwig, K. R. Isoplot/Ex version 2.49: A geochronology toolkit for Microsoft Excel. *Berkeley Geochronology Center Special Publication* **55** (2001).
27 Millard, A. R. & Hedges, R. E. A diffusion-adsorption model of uranium uptake by archaeological bone. *Geochimica et Cosmochimica Acta* **60**, 2139-2152 (1996).
28 Pike, A. & Hedges, R. U-series dating of bone using the diffusion-adsorption model. *Geochimica et Cosmochimica Acta* **66**, 4273-4286 (2002).
29 Sambridge, M., Grün, R. & Eggins, S. U-series dating of bone in an open system: the diffusion-adsorption-decay model. *Quaternary Geochronology* **9**, 42-53 (2012).
30 Jain, M., Murray, A. & Bøtter-Jensen, L. Characterisation of blue-light stimulated luminescence components in different quartz samples: implications for dose measurement. *Radiation Measurements* **37**, 441-449 (2003).
31 Smith, B. W. & Rhodes, E. J. Charge movements in quartz and their relevance to optical dating. *Radiation Measurements* **23**, 329-333 (1994).
32 Wintle, A. G. & Murray, A. S. A review of quartz optically stimulated luminescence characteristics and their relevance in single-aliquot regeneration dating protocols. *Radiation measurements* **41**, 369-391 (2006).
33 Li, B., Jacobs, Z., Roberts, R. G., Galbraith, R. & Peng, J. Variability in quartz OSL signals caused by measurement uncertainties: Problems and solutions. *Quaternary Geochronology* **41**, 11-25 (2017).
34 Fu, X., Cohen, T. J. & Arnold, L. J. Extending the record of lacustrine phases beyond the last interglacial for Lake Eyre in central Australia using luminescence dating. *Quaternary Science Reviews* **162**, 88-110 (2017).
35 Galbraith, R. F. & Roberts, R. G. Statistical aspects of equivalent dose and error calculation and display in OSL dating: An overview and some recommendations. *Quaternary Geochronology* **11**, 1-27 (2012).
36 Lowick, S. E., Preusser, F. & Wintle, A. G. Investigating quartz optically stimulated

luminescence dose–response curves at high doses. *Radiation Measurements* **45**, 975-984 (2010).
37 Murray, A., Buylaert, J.-P., Henriksen, M., Svendsen, J.-I. & Mangerud, J. Testing the reliability of quartz OSL ages beyond the Eemian. *Radiation Measurements* **43**, 776-780 (2008).
38 Arnold, L. J., Bailey, R. M. & Tucker, G. E. Statistical treatment of fluvial dose distributions from southern Colorado arroyo deposits. *Quaternary Geochronology* **2**, 162-167 (2007).
39 Thomsen, K. J. *et al.* Testing single-grain quartz OSL methods using sediment samples with independent age control from the Bordes-Fitte rockshelter (Roches d'Abilly site, Central France). *Quaternary Geochronology* **31**, 77-96 (2016).
40 Bailey, R., Singarayer, J., Ward, S. & Stokes, S. Identification of partial resetting using $D_e$ as a function of illumination time. *Radiation Measurements* **37**, 511-518 (2003).
41 Olley, J. M., Murray, A. & Roberts, R. G. The effects of disequilibria in the uranium and thorium decay chains on burial dose rates in fluvial sediments. *Quaternary Science Reviews* **15**, 751-760 (1996).
42 Murray, A. *et al.* Optically stimulated luminescence dating using quartz. *Nature Reviews Methods Primers* **1**, 72 (2021).
