# Supplementary material for: New Late Pleistocene age for the Homo sapiens skeleton from Liujiang southern China
Source: Nat Commun. 2024 Apr 29;15:3611. doi: 10.1038/s41467-024-47787-3 (PMC11058812; doi:10.1038/s41467-024-47787-3)
Supplement: Supplementary file 3 — Description of Additional Supplementary Files [file 41467_2024_47787_MOESM3_ESM.pdf]

Supplementary Data 1. Trace element concentrations (ppm) for sediments from the Liujiang section and the sediments housing in the medullary cavity of the Liujiang left femur (LJHS).

Supplementary Data 2. MC-ICPMS U-series dating results obtained on the samples from the Tongtianyan cave, with ±2σ absolute uncertainties.

Supplementary Data 3. Concentrations of ^{238}U, ^{232}Th, ^{226}Ra and their decay products, and ^{40}K, as well specific radionuclide activity ratios for the OSL samples from the Liujing section.

Supplementary Data 4. OSL dating results, with uncertainty given at ±1σ level. Age uncertainties include a 2.1% systematic error to allow for any bias associated with calibration of the laboratory beta source.

Supplementary Data 5. OSL and U-Th ages ('Unmodelled age ranges') and corresponding Bayesian age model estimates ('Modelled age ranges') obtained using the OxCal 4.4 platform, at 95.4% probability.

Supplementary Code 1. CQL code for the Bayesian age model.
