# Supplementary material for: New Late Pleistocene age for the Homo sapiens skeleton from Liujiang southern China
Source: Nat Commun. 2024 Apr 29;15:3611. doi: 10.1038/s41467-024-47787-3 (PMC11058812; doi:10.1038/s41467-024-47787-3)
Supplement: Supplementary file 9 — Supplementary Code 1 [file 41467_2024_47787_MOESM9_ESM.docx]

**Supplementary Code 1.** CQL code for the Bayesian age model.

| Plot()  {  Outlier_Model("General",T(5),U(0,4),"t");  Sequence("Liujiang")  {  Boundary("Start Layer 5");  Sequence ("Layer 5")  {  Date("HED-684", N(2022-53200, 6400))  {  z=1433;  Outlier("General", 0.05);  };  Date("HED-683", N(2022-48400, 6000))  {  z=1391;  Outlier("General", 0.05);  };  };  Boundary("Boundary Layers 5/4");  Sequence ("Layer 4")  {  Date("HED-682", N(2022-42300, 5600))  {  Outlier("General", 0.05);  };  };  Boundary("Boundary Layers 4/3");  Phase ("Layer 3")  {  Date("HED-627", N(2022-34100, 4400))  {  Outlier("General", 0.05);  };  Date("HED-642", N(2022-37800, 5400))  {  Outlier("General", 0.05);  };  };  Boundary("End Layer 3");  Boundary("Start Layer 2");  Phase ("Layer 2")  {  Date("HED-628", N(2022-24200, 3200))  {  Outlier("General", 0.05);  };  Date("HED-641", N(2022-24600, 4000))  {  Outlier("General", 0.05);  };  Date("HED-640", N(2022-21800, 3600))  {  Outlier("General", 0.05);  };  R_Date("Beta-653001", 25920, 120)  {  Outlier("General", 0.05);  };  };  Before ()  {  Date("LJHS U-series date", N(2022-22500, 6160))  {  Outlier("General", 0.05);  };  };  Boundary("End Layer 2");  Boundary("Start Layer 1");  Phase ("Layer 1")  {  Date("LJ20-FS2", N(2022-16100, 1800))  {  Outlier("General", 0.05);  };  Date("LJ20-FS1", N(2022-13900, 1400))  {  Outlier("General", 0.05);  };  R_Date("Beta-654800", 11610, 30)  {  Outlier("General", 0.05);  };  R_Date("BA230072", 11135, 50)  {  Outlier("General", 0.05);  };  };  Boundary("End Layer 1");  };  }; |
| --- |
