# Supplementary material for: New Late Pleistocene age for the Homo sapiens skeleton from Liujiang southern China
Source: Nat Commun. 2024 Apr 29;15:3611. doi: 10.1038/s41467-024-47787-3 (PMC11058812; doi:10.1038/s41467-024-47787-3)
Supplement: Supplementary file 10 — Reporting Summary [file 41467_2024_47787_MOESM10_ESM.pdf]

nature portfolio

Corresponding author(s): Qingfeng Shao and Michael D. Petraglia

Last updated by author(s): Apr 9, 2024

Reporting Summary

Nature Portfolio wishes to improve the reproducibility of the work that we publish. This form provides structure for consistency and transparency in reporting. For further information on Nature Portfolio policies, see our Editorial Policies and the Editorial Policy Checklist.

Statistics

For all statistical analyses, confirm that the following items are present in the figure legend, table legend, main text, or Methods section.

n/a	Confirmed	
☐	☒	The exact sample size (*n*) for each experimental group/condition, given as a discrete number and unit of measurement
☐	☒	A statement on whether measurements were taken from distinct samples or whether the same sample was measured repeatedly
☒	☐	The statistical test(s) used AND whether they are one- or two-sided<br>*Only common tests should be described solely by name; describe more complex techniques in the Methods section.*
☒	☐	A description of all covariates tested
☒	☐	A description of any assumptions or corrections, such as tests of normality and adjustment for multiple comparisons
☐	☒	A full description of the statistical parameters including central tendency (e.g. means) or other basic estimates (e.g. regression coefficient) AND variation (e.g. standard deviation) or associated estimates of uncertainty (e.g. confidence intervals)
☒	☐	For null hypothesis testing, the test statistic (e.g. *F*, *t*, *r*) with confidence intervals, effect sizes, degrees of freedom and *P* value noted<br>*Give P values as exact values whenever suitable.*
☐	☒	For Bayesian analysis, information on the choice of priors and Markov chain Monte Carlo settings
☒	☐	For hierarchical and complex designs, identification of the appropriate level for tests and full reporting of outcomes
☒	☐	Estimates of effect sizes (e.g. Cohen's *d*, Pearson's *r*), indicating how they were calculated

Our web collection on statistics for biologists contains articles on many of the points above.

Software and code

Policy information about availability of computer code

Data collection	Microsoft Excel 2016
Data analysis	Oxcal 4.4; Analysize program (https://github.com/greigpaterson/AnalySize); Origin (https://www.originlab.com/); RISO Luminescence Analyst Version 4.31.9; IRM-CLG program (Excel Workbook); Grapher 16.4.432; Microsoft Excel 2016; Matlab 2018

For manuscripts utilizing custom algorithms or software that are central to the research but not yet described in published literature, software must be made available to editors and reviewers. We strongly encourage code deposition in a community repository (e.g. GitHub). See the Nature Portfolio guidelines for submitting code & software for further information.

Data

Policy information about availability of data

All manuscripts must include a data availability statement. This statement should provide the following information, where applicable:

- Accession codes, unique identifiers, or web links for publicly available datasets
- A description of any restrictions on data availability
- For clinical datasets or third party data, please ensure that the statement adheres to our policy

All relevant data and codes are available in the main text and the accompanying supplementary materials. Source data are provided as a Source Data file. The Liujiang human and mammalian fossil remains referred to in this study are currently in Institute of Vertebrate Paleontology and paleoanthropology (IVPP), Chinese Academy of Sciences, Beijing, and a part of mammalian fossil teeth is at Nanjing Normal University, Nanjing, China.

Research involving human participants, their data, or biological material

Policy information about studies with human participants or human data. See also policy information about sex, gender (identity/presentation), and sexual orientation and race, ethnicity and racism.

Reporting on sex and gender	this doses not apply to this study
Reporting on race, ethnicity, or other socially relevant groupings	this doses not apply to this study
Population characteristics	this does not apply to this study
Recruitment	this dose not apply to this study
Ethics oversight	this does not apply to this study

Note that full information on the approval of the study protocol must also be provided in the manuscript.

Field-specific reporting

Please select the one below that is the best fit for your research. If you are not sure, read the appropriate sections before making your selection.

☐ Life sciences ☐ Behavioural & social sciences ☒ Ecological, evolutionary & environmental sciences

For a reference copy of the document with all sections, see nature.com/documents/nr-reporting-summary-flat.pdf

Ecological, evolutionary & environmental sciences study design

All studies must disclose on these points even when the disclosure is negative.

Study description	The study performed detailed sedimentological and geochemical comparisons between the sediments from the Liujiang human fossils and those from various depositional units at the Tongtianyan cave to determine the origin of the human fossils. C-14, U-series and optically stimulated luminescence (OSL) dating of the human and mammalian fossils, and sedimentary sediments were combined to constrain the age range of the human fossils.
Research sample	Cave sedimentary deposits were used for geochemical analyses and OSL dating, and flowstone, human and mammalian fossil remains were used for U-series dating.
Sampling strategy	Eleven sedimentary samples were collected from Units II and III of the Liujiang sequence for OSL dating by hammering steel tubes (20 cm-long cylinders with a diameter of 5 cm) into a freshly dug vertical section, approximately 1kg for each sample. Eight mammalian fossil samples and four flowstone samples were found in situ at the Tongtianyan cave for U-series dating. A total of 17 subsamples was extracted with a dental-drill from the Liujiang left femur for U-series profile analyses. One charcoal sample and two organic sediment samples were collected in situ for C-14 dating. One sediment sample was extracted from the medullary cavity of the Liujiang left femur for geochemical analyses, in the IVPP laboratory.
Data collection	Sedimentary and fossil samples used in the study were manually collected at Tongtianyan cave and IVPP laboratory by J.Y.G., S.X. and Q.F.S. C-14 data were measured by the use of an accelerator mass spectrometer (AMS). U-series, Sr-Nd isotopes and trace element data were measured by the use of inductively-coupled plasma mass spectrometry. Grain-size was determined using a Malvern Mastersizer 3000 particle analyzer. The colour measurements were conducted using a Courtney Minolta CM-400 colorimeter. Luminescence measurements were made on a Risø Model DA-20 TL/OSL reader.
Timing and spatial scale	Field sampling and laboratory analyses started from the autumn of 2019 and finally ended in the winter of 2023.
Data exclusions	No data were excluded from the analysis.
Reproducibility	The results are not from experiments and therefore reproducibility was not tested.
Randomization	This does not apply to this study since no experiment was conducted.
Blinding	This does not apply to this study since no experiment was conducted.

Did the study involve field work? ☒ Yes ☐ No

Field work, collection and transport

Field conditions	The study site is in a subtropical monsoon climate, with annual average temperature of 20.5 degree and rainfall of 1470 mm. The inside of Tongtianyan cave is dark and humid, with some old and active speleothem deposits.
Location	Tongtianyan cave (24°10′59″N, 109°25′56″E, 164 m above mean sea level) is located in the Liujiang District of Liuzhou City, Guangxi Province, southern China.
Access & import/export	Tongtianyan cave can be accessed by public transport, which is approximately 10 km from the center of Liuzhou City, Guangxi Province.
Disturbance	None

Reporting for specific materials, systems and methods

We require information from authors about some types of materials, experimental systems and methods used in many studies. Here, indicate whether each material, system or method listed is relevant to your study. If you are not sure if a list item applies to your research, read the appropriate section before selecting a response.

Materials & experimental systems

n/a	Involved in the study	
☒	☐	Antibodies
☒	☐	Eukaryotic cell lines
☐	☒	Palaeontology and archaeology
☒	☐	Animals and other organisms
☒	☐	Clinical data
☒	☐	Dual use research of concern
☒	☐	Plants

Methods

n/a	Involved in the study	
☒	☐	ChIP-seq
☒	☐	Flow cytometry
☒	☐	MRI-based neuroimaging

Palaeontology and Archaeology

Specimen provenance	Sedimentary samples and mammalian fossil remains were collected in situ at Tongtianyan cave, located in the Liujiang District of Liuzhou City, Guangxi Province. Powder samples of human fossils were collected at IVPP laboratory. Sampling at Tongtianyan cave was permitted by the directors of Lotus Cave Science Museum, Liuzhou, Guangxi Province. Sampling on the human fossils was permitted by the IVPP directors, Chinese Academy of Sciences, Beijing.
Specimen deposition	The Liujiang human fossils and the remains of C-14 and OSL dating samples are currently housed in IVPP, Chinese Academy of Sciences, Beijing. The remains of U-series dating samples are at Nanjing Normal University, Nanjing.
Dating methods	AMS C-14, OSL and U-series dating methods were used in the study. New AMS C-14 dates were obtained on the samples of charcoal and organic sediments collected in situ at Tongtianyan cave. New OSL dates were obtained on the sedimentary deposits at Tongtianyan cave. New U-series dates were obtained on the flowstone, fossil teeth as well as on the human fossils from Tongtianyan cave. AMS C-14 analyses were carried out at the Beta analytic laboratory, US, and at Peking University, China. OSL dating analyses were carried out at Institute of Vertebrate Paleontology and Paleoanthropology, Chinese Academy of Sciences, Beijing, China. U-series dating analyses were carried out at Nanjing Normal University, Nanjing, China. The details of the dating methods are described in the Method and the Supplementary Materials.

☒ Tick this box to confirm that the raw and calibrated dates are available in the paper or in Supplementary Information.

Ethics oversight	Ethics approval or guidance was not required as the study did not involve extant human or animal subjects.

Note that full information on the approval of the study protocol must also be provided in the manuscript.

Plants

Seed stocks	this does not apply in this study
Novel plant genotypes	this dose not apply in this study
Authentication	this dose not apply in this study
